# IL8 and PMA Trigger the Regulation of Different Biological Processes in Granulocyte Activation

**DOI:** 10.3389/fimmu.2019.03064

**Published:** 2020-01-14

**Authors:** Roxane L. Degroote, Maria Weigand, Stefanie M. Hauck, Cornelia A. Deeg

**Affiliations:** ^1^Chair of Physiology, Department of Veterinary Sciences, LMU Munich, Munich, Germany; ^2^Research Unit Protein Science, Helmholtz Center Munich, German Research Center for Environmental Health GmbH, Munich, Germany

**Keywords:** innate immune cell activation, differential proteomics, interleukin 8 (IL8), PMA, LPS, neutrophil, signal transduction, biological process

## Abstract

The molecular mechanisms driving specific regulation of neutrophils are not completely understood to date. In order to characterize fundamental granulocyte features on protein level, we analyzed changes in proteome composition as reaction to stress from cell activation processes. For this purpose, we isolated primary granulocytes from equine whole blood through density gradient centrifugation followed by sodium chloride lysis and stimulated cells for 30 min with interleukin-8 (IL8) due to its role as a chemotactic factor for neutrophils. We additionally used phorbol 12-myristate 13-acetate (PMA) and lipopolysaccharide (LPS), which are primarily associated to neutrophil extracellular trap formation and release of reactive oxygen species. From mass spectrometry analysis, we identified a total of 2,032 proteins describing the whole granulocyte proteome, including 245 proteins (12% of identified proteome) newly associated to *in vivo* expression in primary equine granulocytes (hypothetical proteins). We also found distinct and different changes in protein abundance (ratio ≥ 2) after short stimulation of cells with various stimuli, pointing to rapid and differentiated reaction pattern. IL8 stimulation resulted in increased protein abundance of 58 proteins (3% of proteome), whereas PMA induced changed protein abundance of 207 (10 % of proteome) and LPS of 46 proteins (2% of proteome). Enrichment analyses clearly showed fundamental differences between stimuli, with primary association of IL8 stimulation to processes in immune response, receptor signaling and signal transduction. Top enrichment for PMA on the other hand pointed to vesicle mediated transport and exocytosis. Stimulation with LPS did not result in any significant enrichment. Although we detected 43% overlap of enrichment categories for IL8 and PMA stimulation, indicating that activation of neutrophils with different stimuli partly induces some similar biological processes and pathways, hierarchical clustering showed clear differences in distribution and biological relevance of clusters between the chosen stimuli. Our studies provide novel information on the granulocyte proteome and offer insights into early, differentiated granulocyte reaction to stimuli, which contribute to a better understanding of molecular mechanisms involved in activation and recruitment of neutrophils, through inflammatory stimuli.

## Introduction

Granulocytes have initially been labeled as short-lived, terminally differentiated cells, driving innate immune response through phagocytosis, degranulation, ROS release and, as described more recently, NETosis (1, 2). However, today, neutrophil diversity and plasticity, with many different subpopulations and finely tuned functional features are evident (3–8). Still relatively little is known about specific, differentiated regulation mechanisms in early granulocyte activation involved in subsequent innate immune responses. For this reason, we investigated fundamental granulocyte features by analyzing changes in proteome composition as reaction to cell activation and allocating these changes to different biological processes and pathways in an equine model. In cells from the adaptive immune system, we previously found major differences in regulation of lymphocyte protein expression in autoimmune disease (9–12). Moreover, we detected differences in the granulocyte proteome, with Talin1 as a key player in disease pathogenesis, indicating a role of the innate immune system in lymphocyte-driven autoimmune disease (13, 14). In retrospect, the granulocytes analyzed in these studies most likely represent the subpopulation of low density neutrophils (LDN), which were recently discovered (15). In present study, LDN were excluded from analysis, due to granulocyte isolation protocol. Here, we were especially interested in the impact of initial activation on downstream innate immune response and the pathways switched on in course of activation-induced cell stress in order to provide fundamental knowledge on granulocyte activation mechanisms.

## Materials and Methods

### Sample Processing

The blood used in this study originated from three resident horses of the LMU equine clinic (aged 12, 20, and 21; kept in straw-embedded stalls with daily access to paddocks), which are at the student's disposal for supervised ultrasound- and health assessment training. Health status was assessed by standard clinical routine examinations. No experimental procedures were performed on these horses. Venous whole blood was collected in tubes supplemented with 25.000 I.U. heparin. After rough sedimentation of erythrocytes, PMN were isolated from plasma by density gradient centrifugation (RT, 290 × g, 25 min, brake off) using Ficoll-Paque PLUS separating solution (GE Life Sciences, Freiburg, Germany). Cells were washed gently (4 C, 400 × g, 10 min) in cold PBS (DPBS devoid of CaCl_2_ and MgCl_2_; Gibco/ThermoFisher Scientific, Germany) and remaining erythrocytes were removed by sodium chloride lysis (lysis in 0.2% NaCl, after 30 s addition of equal part 1.6% NaCl to restore isotonicity). Cells were washed (4°C, 400 × g, 10 min) and resuspended in PBS with 0.2% Glucose. From each animal used in the experiment, we prepared aliquot portions of 6 × 10^5^ cells/500 μl. These cell aliquots were separately stimulated with recombinant equine interleukin-8 (IL8; Kingfisher Biotec; 1 ng/ml), phorbol 12-myristate 13-acetate (PMA; Sigma-Aldrich/Merck, Darmstadt, Germany; 5 μg/ml) and lipopolysaccharide (LPS; Sigma-Aldrich/Merck, Darmstadt, Germany; 5 μg/ml) for 30 min in a CO_2_ incubator (37°C, 5% CO_2_). Untreated medium control (mc) was incubated under the same conditions but without stimulating agent. After stimulation, each of the stimulated and mc aliquots was topped up to 1 ml with PBS with 0.2% Glucose and pelleted (4°C, 2,300 × g, 10 min). All Samples were stored at −20°C. Shortly before mass spectrometry analysis, cells were thawed and lysed in urea buffer (8 M urea in 0.1 M Tris/HCl pH 8.5), and protein amount was determined with Bradford protein assay (16).

### Mass Spectrometry Analysis

From each sample, 10 μg total protein was digested with LysC and trypsin by filter-aided sample preparation (FASP) as previously described (17). Acidified eluted peptides were analyzed in the data-dependent mode on a Q Exactive HF mass spectrometer (Thermo Fisher Scientific, Bremen, Germany) online coupled to a UItimate 3000 RSLC nano-HPLC (Dionex). Samples were automatically injected and loaded onto the C18 trap column, eluted after 5 min and separated on the C18 analytical column (75 μm ID × 15 cm, Acclaim PepMAP 100 C18. 100 Å/size, LC Packings, Thermo Fisher Scientific, Bremen, Germany) by a 90 min non-linear acetonitrile gradient at a flow rate of 250 nl/min. MS spectra were recorded at a resolution of 60,000. After each MS1 cycle, the 10 most abundant peptide ions were selected for fragmentation.

### Data Processing

Label-free quantitative analysis was performed using Progenesis QI software (version 2.5, Non-linear Dynamics, Waters, Newcastle upon Tyne, U.K.) as described (18, 19), with raw MS spectral files imported, followed by automatic peak picking and retention time alignment and normalization of total peak intensities across all samples to minimize loading differences. All MS/MS spectra were exported from Progenesis QI software as Mascot generic files (mgf) and searched against Ensembl Horse protein database (version 3.0, http://www.ensembl.org) for peptide identification with Mascot (version 2.5.1). Search parameters used were 10 ppm peptide mass tolerance, 20 mmu fragment mass tolerance, one missed cleavage allowed, carbamidomethylation as fixed modification and methionine oxidation as well as deamidation of asparagine and glutamine as variable modifications. Mascot integrated decoy database search was set to a false discovery rate (FDR) of 1% when searching was performed on the concatenated mgf files with a percolator ion score cut-off of 13 and an appropriate significance threshold *p*. Identifications were re-imported into Progenesis QI and redundancies grouped following the rules of parsimony.

### Data Analysis

Differential protein abundance was determined by comparison of the mean normalized peptide abundance from the extracted ion chromatograms. Proteins were considered differentially expressed at stimulating agent/mc ratio ≥ 2.0. Bioinformatic analysis was performed on human orthologs of gene names from differentially expressed equine proteins with open source software ShinyGO v0.60: http://bioinformatics.sdstate.edu/go60/ (20) with the following settings: search species human, *P*-value cutoff (FDR) 0.05, number of most significant terms to show 30. *P*-value for enrichment analysis was calculated via hypergeometric distribution, followed by correction using FDR. Venn diagram was made with open source tool: http://bioinformatics.psb.ugent.be/webtools/Venn/.

## Results

### Two Thousand Thirty-Two Proteins Describing the Granulocyte Proteome

Using mass spectrometry analysis, we identified the equine whole granulocyte proteome, comprising a total of 2,032 proteins. Among the identifications, we found 245 proteins (hypothetical proteins) which have not been associated to the *in vivo* protein expression repertoire of equine granulocytes so far (Supplemental Table 1). These proteins represent 12% of the total granulocyte proteome identified here.

### Short Stimulation Time of Only 30 min Results in Rapid and Differentiated Reactions of Cells

**Figure 1:**
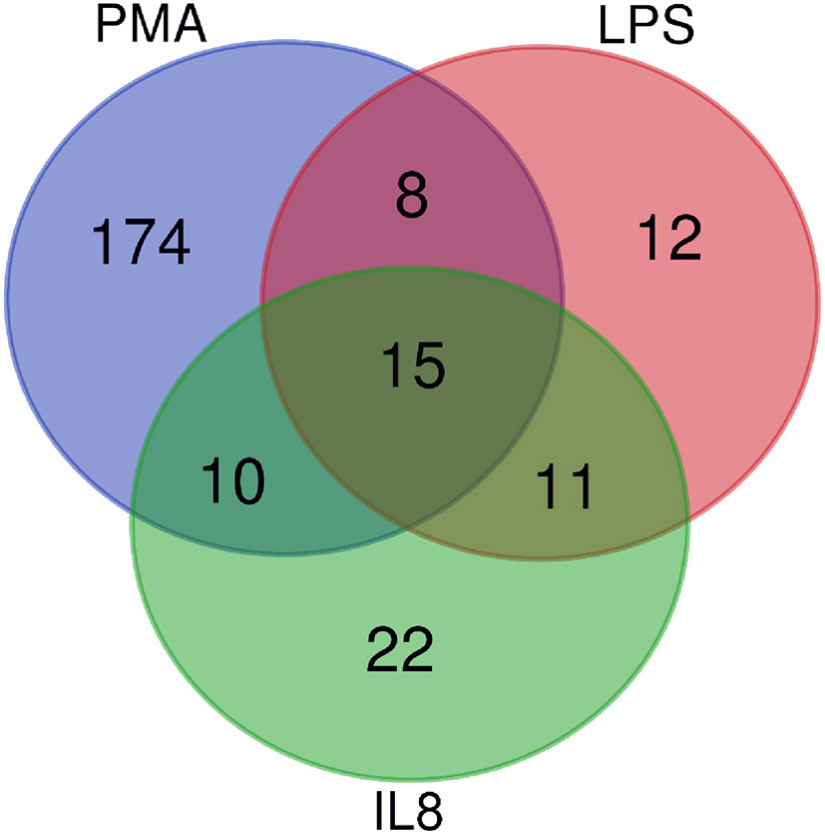
Venn Diagramm of overlapping differentially expressed proteins from IL8, PMA, and LPS stimulated cells. Fifteen proteins are differentially expressed among all stimulation groups.

After stimulation with three different stimulating agents, we found distinct changes in granulocyte protein abundance compared to medium controls (ratio cut-off ≥ 2). In detail, cells stimulated with LPS showed higher expression levels of 46 proteins (2% of proteome), whereas PMA induced increased protein abundance of 207 proteins (10% of proteome). IL8 stimulation resulted in increased protein expression levels of 58 proteins (3% of proteome) (Supplemental Table 2). All of these differentially abundant proteins summed up to a total of 252, from which only 15 showed higher expression levels in all three stimulating agent groups (Figure 1, Table 1). Analysis of differentially expressed proteins per stimulation group revealed 12 unique proteins from LPS and 174 from PMA stimulated cells as well as 22 proteins with unique appearance in cells stimulated with IL8 (Figure 1, Table 1).

**Table 1:** Gene names for shared and unique proteins ≥2 from granulocyte-derived mass spectrometry list.

	**Shared proteins**				**Unique proteins**							
Stimulating agent	IL8	IL8	IL8		IL8							
	PMA	PMA		PMA		PMA						
	LPS		LPS	LPS								LPS
No. of proteins	15	10	11	8	22	174						12
Gene names	AAMDC	DNM1	ALAD	CALCOCO1	ADAMDEC1	A2M	COMT	GYS1	NT5C1A	RIPOR2	SYT5	ACTBL2
	ATP2B1	EXOSC2	ARID1B	EIF4G2	AIFM1	ABHD14B	COPS6	H2AFV	NUCKS1	RNASEL	SYTL3	DCUN1D1
	DMTN	GMPR2	BAX	IWS1	CARHSP1	ACSL4	COX5A	HARS2	NUDT3	RPL12	TACC3	DCXR
	DNASE1L1	JPT1	CPNE6	PROM1	CASP1	ADAM10	CPSF6	HBE1	NUMB	RPL15	TAF2	DHX58
	EEA1	IMPDH2	ECHDC1	RHEB	CDC37	ADD2	CWF19L1	HIST1H1A	NUP210	RPL18	TAOK3	HIKESHI
	ENSA	KCNA10	GNS	SIRPB1	CNP	ADD3	CXorf58	HIST1H3A	NUP62	RPL9	TAPBP	RPS4X
	FARSB	RAP1A	PPP1R18	TBCC	CREG1	ADPGK	CYP2C19	HSD17B12	OAS3	RPN1	TBC1D13	SDHB
	GLYR1	SRSF4	PSMC1	TMEM128	CRYZ	AGPAT2	DCTN3	HSPE1	PFN	RPS6KA2	TEDC1	SEPT11
	HCFC1	VKORC1	SEC23IP		DDOST	ALDH16A1	DENND3	HUWE1	PGRMC1	RPS8	TM9SF2	SLC47A2
	IPCEF1	ZBTB45	SH2D5		DLAT	ARHGAP10	DES	HVCN1	PI4KA	S100A7	TMED10	UBE2H
	LST1		ZNF207		HDLBP	ARL6IP1	DHCR7	IGSF6	PKP1	SARS	TPD52L2	WASHC2A
	PSIP1				IGHG4	ATP6AP1	DNM1L	ILVBL	PPM1F	SEC24A	TRMT112	WDR44
	RPRD1B				IKBKB	ATP8A1	DOCK10	IMMT	PRPF8	SELENOH	TRPC3	
	RPS4X				NAXD	B3GNT2	DOT1L	IRAK3	PSMA7	SEMA3E	TUFM	
	VPS37C				NPEPL1	BAK1	ECHS1	IRF3	PSMB8	SERBP1	UBE2M	
					PAG1	BCAP29	EIF3H	ISG15	PSMB9	SF3A1	UBR4	
					PSMC6	BIN1	EIF4H	ISG20	PSMD7	SLC17A3	UQCRC2	
					PSMD12	BMX	ENOPH1	JAK3	QSOX1	SLC28A1	USP15	
					SARNP	BPGM	EPB41	KARS	R3HCC1	SMAP2	VARS	
					SH3GLB1	BTBD11	ERH	KLF12	RAB43	SNX27	VDAC3	
					SIK3	CAMKK2	ESYT2	LZIC	RASGRP2	SOD2	VPS11	
					VAV2	CARMIL2	FABP5	MCFD2	RBBP4	SPCS2	VPS26A	
						CASP14	GHDC	MCU	RBBP7	SRSF6	VPS28	
						CASS4	GLOD4	MOGS	RBM8A	SSR1	VTI1A	
						CD109	GM2A	MPDU1	RDH16	STK38	WDR5	
						CD300LF	GMFG	MTCH2	RECQL	STRN	WFIKKN1	
						CES2	GNG12	MYADM	RENBP	SYNE1	YARS	
						CHMP3	GRHPR	MYO1E	REXO2	SYNE2	YIF1B	
						COL4A3BP	GRN	NAF1	RHAG	SYPL1	ZSCAN4	

### Reaction of Innate Immune Cells to Different Stimuli Are Respectively Clustered in Three Distinct Networks

In order to understand the association of the differentially expressed proteins to biological processes and their known role in granulocyte activation pathways, we analyzed the data from the 15 proteins present in all groups (Figure 1, Table 1, Supplemental Figure 1, Supplemental Table 3) as well as LPS, PMA, and IL8 groups with open source software ShinyGO. LPS stimulation data did not result in any significant enrichment and clustering of the differentially expressed proteins in these cells. Therefore, we looked into GO category assignment for these proteins and found eight high level categories mainly connected to cell metabolism, intracellular transport and response to stress (Supplemental Table 4). Data from IL8 and PMA stimulated cells, however, revealed three distinct clusters. Comparison of these enrichment category clusters showed a 43% overlap between IL8 and PMA stimulation groups, with neutrophil activation and cellular catabolic processes as the two major shared functional categories (**Figure 3**, **Table 3**). The unique clusters for each stimulant, however, showed a clear difference in reaction of cells to stimuli: PMA stimulated innate immune cells reacted with processes involved in intracellular transportation processes, whereas IL8 stimulated cells showed involvement in signal transduction pathways (**Figure 3**, **Table 3**).

### Unique Reaction of Cells to IL8 Stimulation Associates to Receptor Signaling, Signal Transduction, and Immune Response

**Figure 2:**
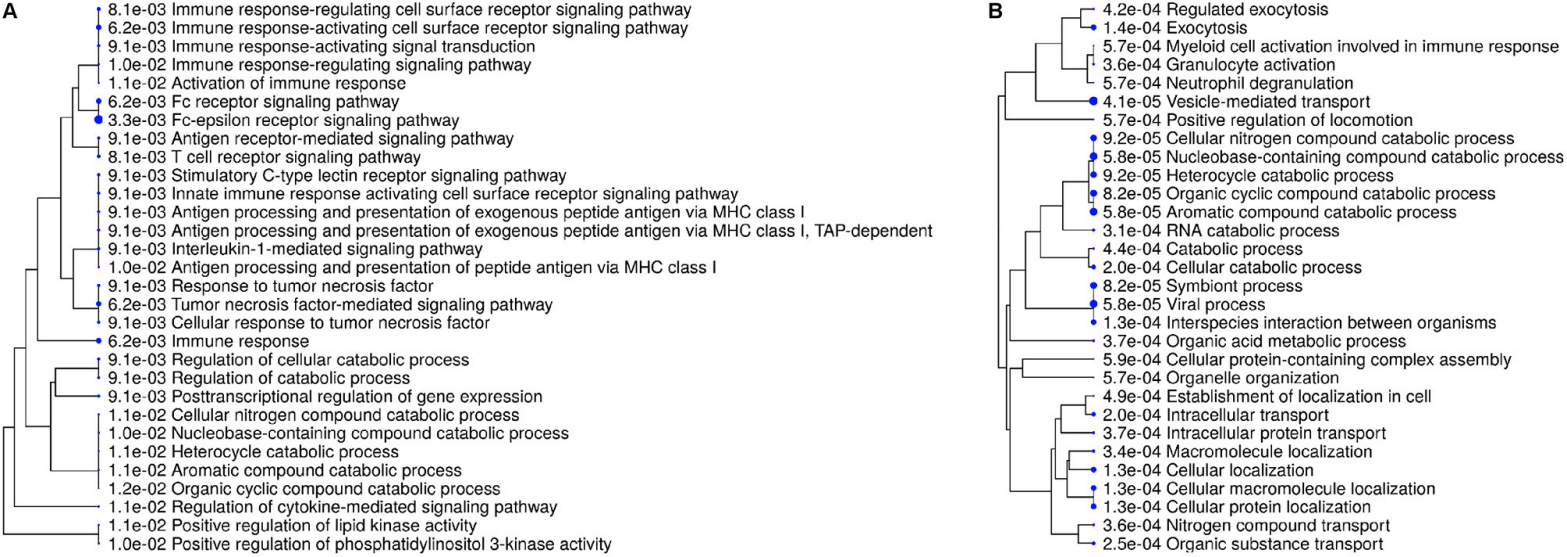
Enrichment tree showing 30 most significant functional categories from biological processes generated from gene names of differentially expressed proteins after stimulation with either IL8 **(A)** or PMA **(B)**. Size of the solid circles corresponds to the enrichment FDR. Proteins used for calculation of enrichment were uniquely present in respective stimulation group.

For more detailed analysis, we subsequently focused on proteins which were differentially abundant in either IL8 or PMA stimulated cells and therefore described as unique for respective stimulus. Hierarchical clustering of enrichment analysis data from unique proteins expressed after PMA stimulation pointed to primary involvement in vesicle-mediated and intracellular transport as well as exocytosis on the one and metabolic processes on the other hand (Figure 2, Table 2). Results from IL8 stimulated cells showed primary association of uniquely expressed proteins to receptor signaling, signal transduction, and immune response with top enrichment for Fc-epsilon receptor and Tumor necrosis factor (TNF) mediated signaling pathways (Figure 2, Table 2). Also, enrichment of PI 3-kinase activity pointed to processes in cytoskeleton dynamics (Figure 2, Table 2). Interestingly, the protein proteasome 26S subunit, ATPase 6 (PSMC6), was allocated to the majority of functional enrichment categories from the IL8 stimulation group (Table 2).

**Table 2:** Enrichment of functional categories describing biological proccesses generated from proteins with differential expression after IL8 and PMA stimulation.

**Enrichment FDR**	**Genes in list**	**Total genes**	**Functional category**	**Genes**
**Biological proccesses from unique Proteins with ratio IL8/mc** **≥** **2**				
0.0033	4	133	Fc-epsilon receptor signaling pathway	PSMC6 IKBKB VAV2 PSMD12
0.0062	10	2,602	Immune response	PAG1 CDC37 CASP1 ADAMDEC1 PSMC6 IKBKB CREG1 VAV2 PSMD12 DDOST
0.0062	4	210	Tumor necrosis factor-mediated signaling pathway	IKBKB CASP1 PSMC6 PSMD12
0.0062	4	203	Fc receptor signaling pathway	PSMC6 IKBKB VAV2 PSMD12
0.0062	5	466	Immune response-activating cell surface receptor signaling pathway	PAG1 PSMC6 IKBKB VAV2 PSMD12
0.0081	4	275	T cell receptor signaling pathway	PAG1 PSMC6 IKBKB PSMD12
0.0081	5	520	Immune response-regulating cell surface receptor signaling pathway	PAG1 PSMC6 IKBKB VAV2 PSMD12
0.0091	6	1,050	Regulation of catabolic process	CARHSP1 SH3GLB1 PSMC6 CDC37 AIFM1 PSMD12
0.0091	5	662	Posttranscriptional regulation of gene expression	CARHSP1 CDC37 SARNP PSMC6 PSMD12
0.0091	6	928	Regulation of cellular catabolic process	CARHSP1 SH3GLB1 PSMC6 CDC37 AIFM1 PSMD12
0.0091	3	141	Innate immune response activating cell surface receptor signaling pathway	PSMC6 IKBKB PSMD12
0.0091	3	135	Stimulatory C-type lectin receptor signaling pathway	PSMC6 IKBKB PSMD12
0.0091	4	368	Response to tumor necrosis factor	IKBKB CASP1 PSMC6 PSMD12
0.0091	3	147	Antigen processing and presentation of exogenous peptide antigen via MHC class I	PSMC6 IKBKB PSMD12
0.0091	3	143	Antigen processing and presentation of exogenous peptide antigen via MHC class I, TAP-dependent	PSMC6 IKBKB PSMD12
0.0091	4	323	Antigen receptor-mediated signaling pathway	PAG1 PSMC6 IKBKB PSMD12
0.0091	5	662	Immune response-activating signal transduction	PAG1 PSMC6 IKBKB VAV2 PSMD12
0.0091	3	118	Interleukin-1-mediated signaling pathway	IKBKB PSMC6 PSMD12
0.0091	4	344	Cellular response to tumor necrosis factor	IKBKB CASP1 PSMC6 PSMD12
0.0102	5	712	Nucleobase-containing compound catabolic process	CARHSP1 AIFM1 CNP PSMC6 PSMD12
0.0102	2	34	Positive regulation of phosphatidylinositol 3-kinase activity	SH3GLB1 VAV2
0.0102	5	714	Immune response-regulating signaling pathway	PAG1 PSMC6 IKBKB VAV2 PSMD12
0.0104	3	167	Antigen processing and presentation of peptide antigen via MHC class I	PSMC6 IKBKB PSMD12
0.0110	2	38	Positive regulation of lipid kinase activity	SH3GLB1 VAV2
0.0110	3	182	Regulation of cytokine-mediated signaling pathway	CDC37 CASP1 IKBKB
0.0110	5	763	Activation of immune response	PAG1 PSMC6 IKBKB VAV2 PSMD12
0.0110	5	765	Cellular nitrogen compound catabolic process	CARHSP1 AIFM1 CNP PSMC6 PSMD12
0.0110	5	764	Heterocycle catabolic process	CARHSP1 AIFM1 CNP PSMC6 PSMD12
0.0114	5	778	Aromatic compound catabolic process	CARHSP1 AIFM1 CNP PSMC6 PSMD12
0.0122	5	813	Organic cyclic compound catabolic process	CARHSP1 AIFM1 CNP PSMC6 PSMD12
**Biological proccesses from unique proteins with ratio PMA/mc** **≥** **2**				
4.15E-05	42	2,220	Vesicle-mediated transport	BCAP29 CHMP3 VPS26A HIST1H1A SYT5 SNX27 VTI1A VPS11 VPS28 TMED10 RAB43 SEC24A ATP6AP1 DNM1L DENND3 ESYT2 NUMB BIN1 SYTL3 MCFD2 MYO1E CD300LF TAPBP GRN PKP1 HUWE1 PGRMC1 PSMD7 QSOX1 HVCN1 ATP8A1 UBR4 GMFG DCTN3 ADAM10 S100A7 CD109 FABP5 GHDC AGPAT2 A2M GM2A
5.76E-05	22	778	Aromatic compound catabolic process	COMT RBM8A NUDT3 ISG20 NT5C1A RNASEL ADPGK BPGM PKP1 NAF1 SERBP1 RPL18 PSMA7 PSMD7 NUP210 RPS8 RPL9 RPL15 RPL12 PSMB8 NUP62 PSMB9
5.76E-05	21	712	Nucleobase-containing compound catabolic process	RBM8A NUDT3 ISG20 NT5C1A RNASEL ADPGK BPGM PKP1 NAF1 SERBP1 RPL18 PSMA7 PSMD7 NUP210 RPS8 RPL9 RPL15 RPL12 PSMB8 NUP62 PSMB9
5.76E-05	25	951	Viral process	OAS3 CHMP3 RNASEL KARS PSMA7 EIF4H IRF3 UBR4 BIN1 COPS6 ISG15 PSMB8 NUP62 PSMB9 NUCKS1 ISG20 RAB43 PI4KA RPL18 NUP210 RPS8 VPS28 RPL9 RPL15 RPL12
8.17E-05	22	813	Organic cyclic compound catabolic process	COMT RBM8A NUDT3 ISG20 NT5C1A RNASEL ADPGK BPGM PKP1 NAF1 SERBP1 RPL18 PSMA7 PSMD7 NUP210 RPS8 RPL9 RPL15 RPL12 PSMB8 NUP62 PSMB9
8.17E-05	25	1,024	Symbiont process	OAS3 CHMP3 RNASEL KARS PSMA7 EIF4H IRF3 UBR4 BIN1 COPS6 ISG15 PSMB8 NUP62 PSMB9 NUCKS1 ISG20 RAB43 PI4KA RPL18 NUP210 RPS8 VPS28 RPL9 RPL15 RPL12
9.22E-05	21	765	Cellular nitrogen compound catabolic process	RBM8A NUDT3 ISG20 NT5C1A RNASEL ADPGK BPGM PKP1 NAF1 SERBP1 RPL18 PSMA7 PSMD7 NUP210 RPS8 RPL9 RPL15 RPL12 PSMB8 NUP62 PSMB9
9.22E-05	21	764	Heterocycle catabolic process	RBM8A NUDT3 ISG20 NT5C1A RNASEL ADPGK BPGM PKP1 NAF1 SERBP1 RPL18 PSMA7 PSMD7 NUP210 RPS8 RPL9 RPL15 RPL12 PSMB8 NUP62 PSMB9
0.0001	36	1,981	Cellular protein localization	HUWE1 VPS11 BCAP29 TBC1D13 SPCS2 VPS26A TM9SF2 UQCRC2 SNX27 VTI1A VPS28 TMED10 ARL6IP1 RAB43 NUP62 SYNE2 SYNE1 DNM1L RIPOR2 SEC24A ADAM10 SYTL3 PPM1F MTCH2 NUMB EPB41 MYADM RPL18 SRSF6 SSR1 NUP210 RPS8 RPL9 RPL15 RPL12 RBM8A
0.0001	48	3,087	Cellular localization	HUWE1 VPS11 BCAP29 DNM1L TBC1D13 COL4A3BP CHMP3 SPCS2 VPS26A TM9SF2 SYT5 UQCRC2 SNX27 VTI1A VPS28 TMED10 ARL6IP1 RAB43 NUP62 SYNE2 SEC24A SYNE1 TRPC3 BAK1 ATP6AP1 DENND3 RIPOR2 ESYT2 NUMB BIN1 ADAM10 SYTL3 PPM1F MTCH2 CPSF6 EPB41 MYADM RPL18 SRSF6 SSR1 NUP210 DCTN3 RPS8 RPL9 RPL15 MCFD2 RPL12 RBM8A
0.0001	25	1,084	Interspecies interaction between organisms	OAS3 CHMP3 RNASEL KARS PSMA7 EIF4H IRF3 UBR4 BIN1 COPS6 ISG15 PSMB8 NUP62 PSMB9 NUCKS1 ISG20 RAB43 PI4KA RPL18 NUP210 RPS8 VPS28 RPL9 RPL15 RPL12
0.0001	36	1,993	Cellular macromolecule localization	HUWE1 VPS11 BCAP29 TBC1D13 SPCS2 VPS26A TM9SF2 UQCRC2 SNX27 VTI1A VPS28 TMED10 ARL6IP1 RAB43 NUP62 SYNE2 SYNE1 DNM1L RIPOR2 SEC24A ADAM10 SYTL3 PPM1F MTCH2 NUMB EPB41 MYADM RPL18 SRSF6 SSR1 NUP210 RPS8 RPL9 RPL15 RPL12 RBM8A
0.0001	24	1,023	Exocytosis	SYT5 VPS11 ATP6AP1 DNM1L SYTL3 TMED10 GRN PKP1 HUWE1 PGRMC1 PSMD7 QSOX1 HVCN1 ATP8A1 UBR4 GMFG ADAM10 S100A7 CD109 FABP5 GHDC AGPAT2 A2M GM2A
0.0002	35	1,959	Intracellular transport	HUWE1 VPS11 BCAP29 TBC1D13 COL4A3BP CHMP3 SPCS2 VPS26A SYT5 UQCRC2 SNX27 VTI1A VPS28 TMED10 ARL6IP1 RAB43 NUP62 SEC24A SYNE2 DNM1L DENND3 SYTL3 CPSF6 BIN1 RPL18 SRSF6 SSR1 NUP210 DCTN3 RPS8 RPL9 RPL15 MCFD2 RPL12 RBM8A
0.0002	41	2,500	Cellular catabolic process	HUWE1 DNM1L COMT PSMA7 PSMD7 UBR4 ECHS1 VPS28 CYP2C19 PSMB8 HBE1 PSMB9 RBM8A NUDT3 CAMKK2 ISG20 RENBP DENND3 NT5C1A USP15 RNASEL ADPGK VPS11 BPGM ISG15 GM2A PKP1 QSOX1 NAF1 VTI1A SERBP1 RPL18 CHMP3 VPS26A NUP210 RPS8 RPL9 FABP5 RPL15 RPL12 NUP62
0.0003	46	3,011	Organic substance transport	HUWE1 VPS11 TBC1D13 COL4A3BP CHMP3 SPCS2 VPS26A ATP8A1 SLC17A3 SYT5 UQCRC2 SNX27 VTI1A SLC28A1 VPS28 TMED10 ARL6IP1 RAB43 NUP62 KARS DNM1L RHAG ACSL4 BCAP29 SEC24A ESYT2 IRF3 NUP210 RNASEL SYTL3 FABP5 MCFD2 GM2A RBM8A PPM1F CPSF6 MCU RPL18 ATP6AP1 VDAC3 SRSF6 SSR1 RPS8 RPL9 RPL15 RPL12
0.0003	15	465	RNA catabolic process	RBM8A ISG20 RNASEL PKP1 NAF1 SERBP1 RPL18 PSMA7 PSMD7 RPS8 RPL9 RPL15 RPL12 PSMB8 PSMB9
0.0003	49	3,354	Macromolecule localization	HUWE1 NAF1 VPS11 BCAP29 TBC1D13 COL4A3BP CHMP3 SPCS2 VPS26A ATP8A1 TM9SF2 UQCRC2 SNX27 VTI1A VPS28 TMED10 ARL6IP1 RAB43 NUP62 SYNE2 KARS DNM1L SYNE1 ACSL4 RIPOR2 SEC24A ESYT2 IRF3 NUP210 ADAM10 SYTL3 MCFD2 GM2A RBM8A PPM1F MTCH2 CPSF6 NUMB MCU EPB41 MYADM RPL18 ATP6AP1 SRSF6 SSR1 RPS8 RPL9 RPL15 RPL12
0.0004	41	2,592	Nitrogen compound transport	HUWE1 VPS11 TBC1D13 RHAG COL4A3BP CHMP3 SPCS2 VPS26A SLC17A3 SYT5 UQCRC2 SNX27 VTI1A SLC28A1 VPS28 TMED10 ARL6IP1 RAB43 NUP62 KARS DNM1L BCAP29 TAPBP SEC24A IRF3 NUP210 SYTL3 MCFD2 RBM8A PPM1F CPSF6 MCU RPL18 ATP6AP1 VDAC3 SRSF6 SSR1 RPS8 RPL9 RPL15 RPL12
0.0004	17	603	Granulocyte activation	KARS GRN PKP1 HUWE1 PGRMC1 PSMD7 QSOX1 HVCN1 ATP8A1 UBR4 GMFG ADAM10 S100A7 FABP5 GHDC AGPAT2 GM2A
0.0004	25	1,194	Intracellular protein transport	HUWE1 VPS11 TBC1D13 SPCS2 VPS26A UQCRC2 SNX27 VTI1A VPS28 TMED10 ARL6IP1 RAB43 NUP62 BCAP29 SEC24A SYTL3 RPL18 SRSF6 SSR1 NUP210 RPS8 RPL9 RPL15 RPL12 RBM8A
0.0004	26	1,276	Organic acid metabolic process	SARS KARS VARS HARS2 ECHS1 YARS ENOPH1 CYP2C19 GRHPR CES2 ACSL4 COMT RENBP HSD17B12 ADPGK FABP5 BPGM SLC17A3 PSMA7 PSMD7 ABHD14B NUP210 B3GNT2 PSMB8 NUP62 PSMB9
0.0004	21	901	Regulated exocytosis	SYT5 DNM1L TMED10 GRN PKP1 HUWE1 PGRMC1 PSMD7 QSOX1 HVCN1 ATP8A1 UBR4 GMFG ADAM10 S100A7 CD109 FABP5 GHDC AGPAT2 A2M GM2A
0.0004	43	2,825	Catabolic process	HUWE1 DNM1L COMT PSMA7 PSMD7 UBR4 ECHS1 VPS28 CYP2C19 PSMB8 HBE1 PSMB9 RBM8A NUDT3 CAMKK2 ISG20 IRAK3 RENBP DENND3 NT5C1A USP15 RNASEL ADPGK VPS11 BPGM ISG15 GM2A PKP1 QSOX1 NAF1 VTI1A SERBP1 RPL18 CHMP3 VPS26A NUP210 RPS8 RPL9 FABP5 CES2 RPL15 RPL12 NUP62
0.0005	38	2,364	Establishment of localization in cell	HUWE1 VPS11 BCAP29 TBC1D13 COL4A3BP CHMP3 SPCS2 VPS26A SYT5 UQCRC2 SNX27 VTI1A VPS28 TMED10 ARL6IP1 RAB43 NUP62 SEC24A TRPC3 BAK1 SYNE2 DNM1L DENND3 NUMB SYTL3 CPSF6 BIN1 RPL18 SRSF6 SSR1 NUP210 DCTN3 RPS8 RPL9 RPL15 MCFD2 RPL12 RBM8A
0.0006	16	577	Neutrophil degranulation	GRN PKP1 HUWE1 PGRMC1 PSMD7 QSOX1 HVCN1 ATP8A1 UBR4 GMFG ADAM10 S100A7 FABP5 GHDC AGPAT2 GM2A
0.0006	17	640	Myeloid cell activation involved in immune response	KARS GRN PKP1 HUWE1 PGRMC1 PSMD7 QSOX1 HVCN1 ATP8A1 UBR4 GMFG ADAM10 S100A7 FABP5 GHDC AGPAT2 GM2A
0.0006	16	576	Positive regulation of locomotion	SEMA3E KARS S100A7 CARMIL2 SYNE2 RIPOR2 ATP8A1 PPM1F CHMP3 NUMB GRN CASS4 PFN1 SOD2 ADAM10 MYADM
0.0006	55	4,098	Organelle organization	RBBP4 HUWE1 VPS11 RECQL TACC3 SYNE2 ARHGAP10 ADD2 PKP1 DNM1L CASS4 DOT1L H2AFV PFN1 HIST1H1A GMFG UQCRC2 ADD3 VTI1A EPB41 TMED10 WDR5 RPL12 CAMKK2 BAK1 GRN PPM1F SEC24A SYNE1 USP15 SERBP1 CARMIL2 ARL6IP1 HIST1H3A NUCKS1 RPS6KA2 VDAC3 RBBP7 COL4A3BP BIN1 SEMA3E CHMP3 IMMT NAF1 RAB43 DES MYADM NUP62 SOD2 ZSCAN4 TAPBP DCTN3 VPS28 COPS6 MCFD2
0.0006	25	1,253	Cellular protein-containing complex assembly	ADD2 DNM1L EIF4H PFN1 SRSF6 GMFG NAF1 ADD3 VPS11 PRPF8 RPL12 SF3A1 CPSF6 SEC24A CHMP3 CARMIL2 RBBP4 HIST1H3A HIST1H1A BIN1 EIF3H TAPBP MYADM RBBP7 TMED10

*Proteins used were uniquely expressed in each stimulation group*.

## Discussion

Knowledge about the molecular mechanisms involved in specific granulocyte activation and subsequent choice of pathways depending on different stressors is still incomplete to date. Moreover, in the past, granulocytes have frequently been underestimated in their ability to execute distinct heterogenic reactions rather than uniform response cascades to any, mainly pathogen-induced stimulus. The past decade has yielded more details on granulocyte heterogeneity and function, not only for processes in the innate immune system but also for regulatory involvement in adaptive immune responses (4, 21). Nevertheless, there are still many signaling processes in granulocyte activation, which need clarification. To gain deeper insight into these processes and to find possible downstream reaction differences between initiating stimuli, we performed a short stimulation assay with freshly obtained, primary equine granulocytes. PMA and LPS, were used as universal stimuli. PMA induces exocytosis, ROS release and NET formation through direct activation of protein kinase C (PKC) and subsequent signal transduction cascade (22). LPS triggers similar responses by binding to TLR4 on neutrophils (23). For specific activation of granulocytes, we used IL8. This cytokine is expressed by a variety of cells, such as monocytes, fibroblasts and endothelial cells and acts as a potent chemoattractant for granulocytes, inducing neutrophil recruitment and chemotaxis via chemokine receptors CXCR1 and CXCR2 (24). Granulocyte activation and identification of resulting differences in behavior, gene regulation and protein expression have previously been performed in other uncommon models such as cattle (25) and pigs (26). Predominantly, studies focus on human granulocytes, however, most of these studies concentrate on one particular morphologic (granules, membrane proteins) (27–29) or functional (NET formation) (30, 31) feature of granulocytes. Few studies describe stimulatory experiments and their effect on the whole granulocyte proteome (32, 33). Compared to these studies, we chose a very short stimulation time in order to detect early proteome changes with possibly transient character. Also, we did not separate the proteome via 2D prior to mass spectrometry analysis.

From our initial proteomics experiment, we unraveled the equine granulocyte proteome, detecting 2,032 proteins (Supplemental Table 1). Similar proteomic based studies have been performed on human granulocytes (34), granulocytes from other species such as cattle (35) and rats (36), as well as neutrophil-associated BALF proteins in horses (37). However, to our knowledge, the full equine neutrophil proteome has not been described to date. Interestingly, 12% of the identified proteins in our study were classified as “hypothetical proteins,” whose existence is predicted, but experimental evidence for *in vivo* expression is lacking. With our studies, we could confirm actual *in vivo* expression of these proteins, associating them to primary granulocyte proteome in horses. We chose equine granulocytes to conduct our experiments, because the equine and human immune system share a wide range of similarities both in granulocyte-lymphocyte ratio, composition and function (38–40). Furthermore, the horse is prone to allergies and autoimmune diseases, which are similarly found in humans (41–45) and adaptive as well as innate immune cells from horses have proven to be valuable tools for studying human diseases (37, 42, 44, 46). Despite certain differences between human and horse neutrophils (47, 48), the horse is still a very promising model, especially for processes and diseases which are not adequately addressed by rodent models. However, more investigations are needed to determine its exact and true translational value, which we provide a basis for with our studies.

**Figure 3:**
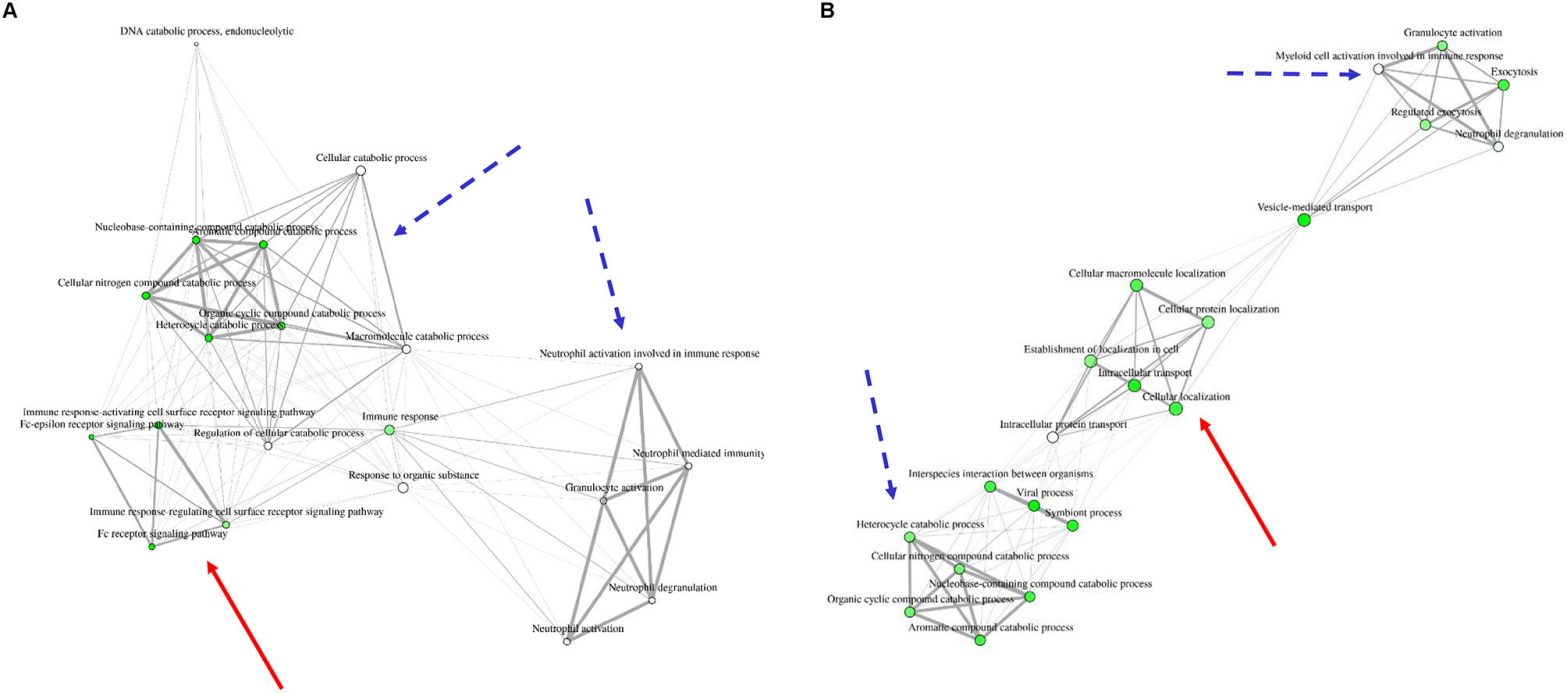
Network clustering for biological processes to which differentially expressed proteins from IL8 **(A)** and PMA **(B)** stimulation were appointed. Three distinct clusters are visible in each stimulation group. Two clusters show similarities between stimulants (dotted blue arrow: granulocyte activation and metabolic processes) whereas one cluster is unique for each group [red arrow: immune response signaling pathways in IL8 stimulated cells **(A)** and cellular protein localization in PMA stimulated cells **(B)**]. For a more clear presentation of clusters, we searched 20 most significant categories instead of 30.

Among all identified proteins, we found a total of 252 differentially abundant proteins after cell stimulation with different stimuli (Supplemental Table 2). Fifteen of these proteins showed higher expression levels in all three stimulating agent groups (Figure 1, Table 1, Supplemental Figure 1, Supplemental Table 3), indicating onset of some mutual reactions to the different stimuli. A larger number of unique proteins with differential expression per stimulant, however, pointed to predominantly differentiated reactions to the different stimuli (Figure 1, Table 1). Further assessment of all differentially abundant proteins from PMA and IL8 samples with ShinyGO enrichment analysis revealed 57% unique network clustering for each stimulant, respectively (Figure 3, Table 3). This shows the ability of granulocytes to distinguish between stimuli and regulate specific pathways in response to selective cell-stressors, although partial immune response is executed independent of stimulation type. Subsequent analysis of solely those proteins that changed abundance uniquely after either IL8 or PMA stimulation highlighted their association to stimulant-characteristic reactions, such as exocytosis and degranulation after PMA stimulation (22), and cytoskeleton dynamics after stimulation with IL8 (49, 50) (Figure 2, Table 2).

**Table 3:** Shared and unique functional categories generated from differentially expressed proteins after IL8 and PMA stimulation.

	**Functional categories from biological process IL8 and PMA**		
Stimulating agent	Unique	Shared	Unique
	IL8	IL8 /PMA	PMA
No. of categories	17	13	17
Functional categories	Antigen receptor-mediated signaling pathway	Aromatic compound catabolic process	Cellular localization
	Cell activation	Cellular catabolic process	Cellular macromolecule localization
	Cellular response to mineralocorticoid stimulus	Cellular nitrogen compound catabolic process	Cellular protein localization
	DNA catabolic process	Granulocyte activation	Establishment of localization in cell
	DNA catabolic process, endonucleolytic	Heterocycle catabolic process	Exocytosis
	Fc receptor signaling pathway	Leukocyte degranulation	Interspecies interaction between organisms
	Fc-epsilon receptor signaling pathway	Myeloid cell activation involved in immune response	Intracellular protein transport
	Immune response	Neutrophil activation	Intracellular transport
	Immune response-activating cell surface receptor signaling pathway	Neutrophil activation involved in immune response	Macromolecule localization
	Immune response-regulating cell surface receptor signaling pathway	Neutrophil degranulation	Nitrogen compound transport
	Macromolecule catabolic process	Neutrophil mediated immunity	Organic substance transport
	MRNA metabolic process	Nucleobase-containing compound catabolic process	Regulated exocytosis
	Regulation of cellular catabolic process	Organic cyclic compound catabolic process	Regulation of biological quality
	Regulation of mRNA stability		RNA catabolic process
	Regulation of RNA stability		Symbiont process
	Response to organic substance		Vesicle-mediated transport
	Tumor necrosis factor-mediated signaling pathway		Viral process

IL8 stimulation yielded the identification of proteasome 26S subunit, ATPase 6 (PSMC6), which showed higher abundance unique to this stimulant (IL8/mc ratio 2.1; *p* < 0.001). PSMC6 is an ATP-dependent proteolytic complex responsible for ubiquitin-dependent protein degradation (51, 52), which is an important regulator of the majority of cellular activity and homeostasis (53). Divergent levels of proteasome activity have a strong impact on disease pathogenesis of several diseases and are used as drug targets in disease treatment (51, 54–56). Thus, the higher abundance of PSMC6 in IL8 stimulated cells might indicate activation of the proteasome in granulocytes with functional importance in downstream regulation of immune response to stress. Subsequent analysis of proteomic data from IL8 stimulated cells revealed that PSMC6 was present in the majority of functional enrichment clusters from biological processes, including the top enriched functional categories tumor necrosis factor (TNF) mediated signaling and Fc-epsilon receptor pathways (Table 2). These two pathways are essential for signal transduction in cells, with a wide functional variety of downstream responses such as apoptosis but also immune and inflammatory responses as well as cell survival, activation and differentiation (57, 58). Interestingly, occurrence of Fc receptors on granulocytes have initially been described as a marker of neutrophil heterogeneity rather than a necessity for optimal neutrophil aggregation and adhesion (59). Especially Fc-epsilon receptor signaling is only present in neutrophils under certain conditions and their exact role is still discussed among experts, whereas other Fc receptor types, such as low-affinity Fc-gamma receptors, are commonly expressed on granulocytes playing an important role in immune complex mediated activation of neutrophils through their downstream pathways (58). Furthermore, Fc receptors are unlikely to mediate PMA-induced cell activation (59), which is consistent with our findings on PMA-stimulated granulocytes, where we found no allocation of uniquely expressed proteins to Fc receptor signaling pathways (Figures 2, 3, Tables 2, 3).

Our findings undermine the ongoing appreciation of granulocyte function toward finely tuned, heterogeneous, specific reactions of more than one subpopulation of neutrophils (4, 7, 8, 60–63). Furthermore, our data shows that a stimulation time of only 30 min is sufficient to initiate substantial and specific changes in granulocyte proteome as reaction to individual stimulating agents (Figure 1, Table 1, Supplemental Table 2). These rapid changes most likely occur due to gene induction of early responding genes but may also be the result of posttranslational modifications mediated by proteins that are activated early in neutrophil responses to stimuli, such as phosphatidylinositol 3-kinase (PI 3-kinase). Interestingly, PI 3-kinase activity appears as functional category from enrichment analysis of our IL8 data (Figure 2A, Table 2), supporting the involvement of PI 3-kinase in IL8-induced protein changes. With its ability to phosphorylate molecules acting as second messengers and thereby switch on downstream intracellular signaling (64), and its involvement in neutrophil chemokinesis and phagosome formation (49, 50, 65), PI 3-kinase merits further investigations in future functional studies. No matter the origin of the changed granulocyte protein repertoire described in our data, it gives insight into early onset of granulocyte activation on protein level, which may be useful to modulate granulocyte mediated pathological processes in future functional experiments. However, more experiments are needed, not only for determination of minimal stimulation times triggering regulation of protein expression levels in granulocytes, but also for analysis of expression kinetics in course of longer stimulation assays. From other comprehensive studies on equine neutrophils we know, that neutrophil extracellular traps (NETs) readily occur in response to adequate stimuli (66) as opposed to cells from other animal models (67). Our protein data however, lack association to this process (Figure 2, Table 2). We assume that the expression differences of proteins associated to NET formation occur after longer stimulation time, as recently described (66). Therefore, increasing the stimulation time in these assays could address protein repertoire changes associated to NET-relevant biological processes such as DNA decondensation, histone citrullination, and related signal transduction. Also, we would expect more prominent clustering of IL8 induced protein changes to cytoskeleton dynamics involved in chemotaxis and phagosome formation, as functional answers of cells to stimuli fluctuate over time (68). Keeping in mind the dynamic character of protein expression patterns in course of cell activation, our data put a spotlight merely on the first reaction to stimuli. This is a very interesting time point in our opinion, because it shows the initiating functional answers in activated cells, which are potentially accessible to experimental modulation. Adding proteomic data from more stimulation times would give a more precise insight into dynamic whole-cell proteome changes throughout the activation process of granulocytes, similar to previous analysis of pre-determined cytokines and degranulation markers by kinetic flow cytometry (69), which needs to be addressed in future studies.

## Conclusion

With our data we provide a fundamental study on activation of primary granulocytes and regulation of downstream immune response by showing that different stimuli provoke divergent and rapid downstream responses through regulation of protein expression in these cells. These expression differences show involvement in various different pathways and biological processes which, among some similarities, differ between stimuli and support knowledge on heterogeneity of granulocytes and their highly selective response to stimuli. The presented data may therefore act as a guide for further, in-depth research on granulocyte response patterns and behavior in health and disease.

## Data Availability Statement

The mass spectrometry proteomics data have been deposited to the ProteomeXchange Consortium via the PRIDE (70) partner repository with the dataset identifier PXD013648.

## Ethics Statement

Collection of blood was permitted by the local authority, Regierung von Oberbayern (Permit number: ROB-55.2Vet-2532.Vet_03-17-88).

## Author Contributions

CD conceived and designed the experiments. RD and SH performed the experiments. RD, SH, MW, and CD analyzed the data. RD wrote the manuscript. All authors critically read the manuscript and approved the final version to be published.

### Conflict of Interest

The authors declare that the research was conducted in the absence of any commercial or financial relationships that could be construed as a potential conflict of interest.
