# Supplementary figures and images for: IL8 and PMA Trigger the Regulation of Different Biological Processes in Granulocyte Activation
Source: Front Immunol. 2020 Jan 14;10:3064. doi: 10.3389/fimmu.2019.03064 (PMC6973177; doi:10.3389/fimmu.2019.03064)

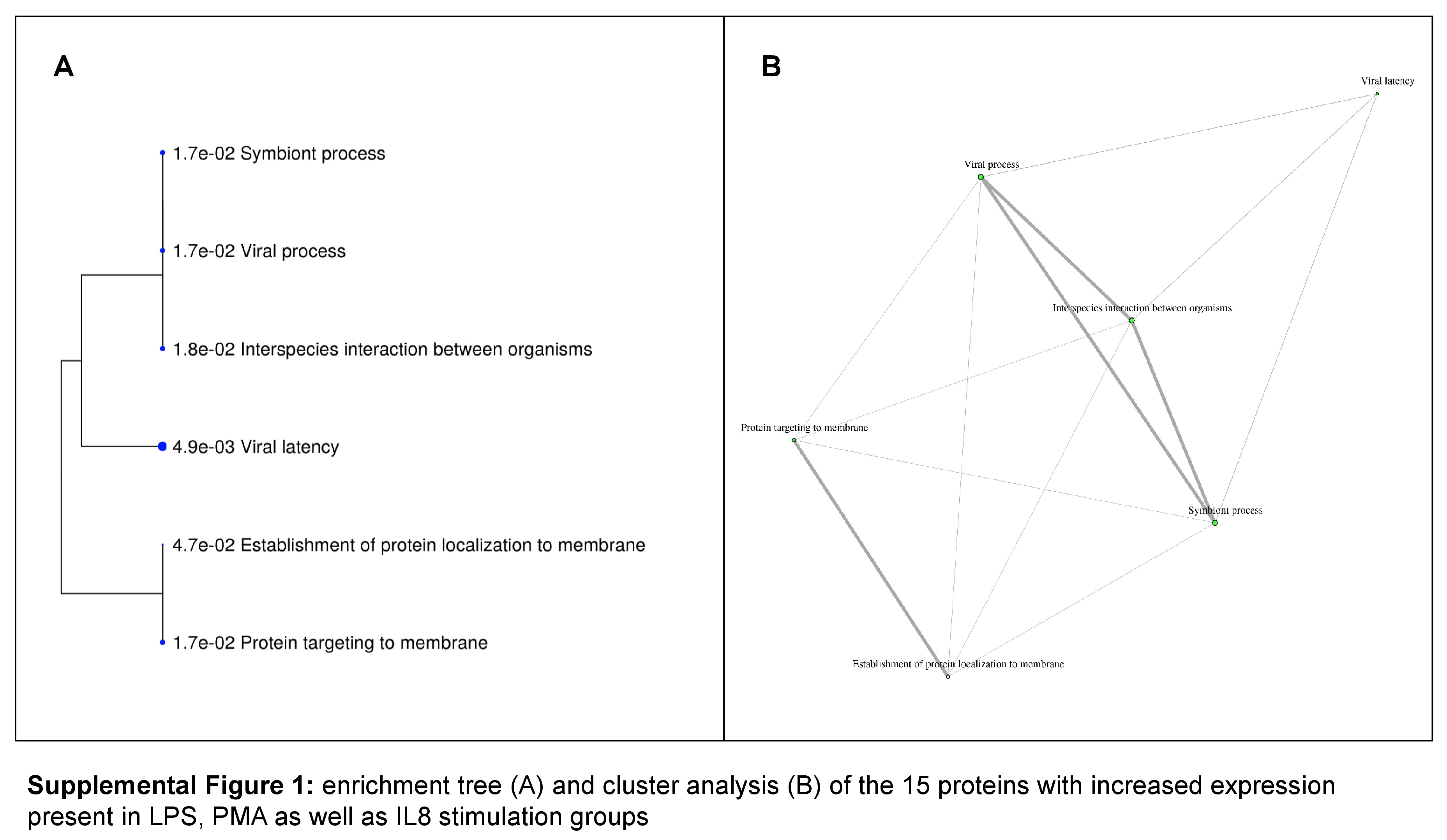

Supplement: Supplementary file 5 [file Image_1.TIFF]
